# Biological Warfare in the 17th Century

**DOI:** 10.3201/eid2209.152073

**Published:** 2016-09

**Authors:** W. Seth Carus

**Affiliations:** National Defense University, Washington, DC, USA

**Keywords:** biological weapons, Candia, Ottoman Empire, plague, Yersinia pestis, Republic of Venice, Russia, bacteria, zoonosis, bioterrorism

**To the Editor:** In an article that reviews evidence of a plot to use plague to break the siege of Candia during the Venetian–Ottoman War of the 17th century, Dr. Thalassinou and her colleagues (*1*) identify an incident previously unknown to historians of biological warfare. However, the authors’ effort to broaden the context for biological weaponry is undermined by a reference to an often repeated allegation for which no credible evidence exists: namely, that during a siege occurring in the Swedish–Russian War of 1710, the Russians catapulted bodies of plague victims into the Swedish-held city of Reval.

Danish historian Karl-Erik Frandsen conducted a careful study of the plague outbreak affecting the Baltic area during 1709–1713 and found no evidence to support this allegation (*2*). Plague was first detected in Reval on August 10, 1710, while the army from Russia was still approaching the city. Reval was not besieged, and the Russians merely camped outside the city while attempting to isolate it. The army dumped corpses into a stream that flowed into Reval, but evidence does not show that the dead were plague victims, nor does evidence exist that clarifies whether the intent was contamination of the water supply or disposal of bodies. Original accounts provide no evidence to suggest that Russians hurled bodies into the city, much less plague-infected bodies. Frandsen estimates that about three quarters of the 20,000 persons in Reval died during the outbreak (*2*).

Intentional introduction of disease has been rare (*3*). Consequently, the incident identified by Thalassinou and her colleagues arouses readers’ interest and inspires speculation.
